# Endovascular Treatment of Intracranial Pial–Dural Arteriovenous Fistula: A Case Report

**DOI:** 10.1055/s-0040-1712533

**Published:** 2020-06-16

**Authors:** D. V. Shchehlov, S. V. Konotopchyk, O. E. Svyrydiuk, I. M. Bortnik, M.Y. Momonova, M.B. Vyval

**Affiliations:** 1 Department of Endovascular Neuroradiology, State Organization “Scientific - Practical Center of Endovascular Neuroradiology NAMS of Ukraine,” Kyiv, Ukraine

**Keywords:** intracranial pial arteriovenous fistula, dural arteriovenous fistula, endovascular occlusion of arteriovenous fistula

## Abstract

Intracranial pial arteriovenous fistula (PAVF) is a rare cerebrovascular pathology characterized by abnormal direct high-flow connection between the pial or cortical feeding artery and draining vein. Dural arteriovenous fistula (DAVF) is a pathological shunt between the meningeal arteries and dural sinuses or meningeal veins. In case of association between PAVF and DAVF, diagnosis and treatment are more challenging. The high-flow arteriovenous shunt and deep venous drainage make PAVF more preferable for endovascular treatment; however, their embolization during single-session procedures can lead to extensive thrombosis of the draining veins and unfavorable outcomes. We present a case report of endovascular embolization of an intracranial PAVF–DAVF in a 2.5-year-old child. At the time of admission, the patient had hydrocephalus, mental retardation, pyramidal insufficiency, and seizures. Occlusion of the fistula was performed during two stages of embolization to reduce the risk of severe venous stasis and venous thrombosis. Guglielmi detachable coils (GDCs) and a liquid embolic agent (Histoacryl with Lipiodol) were used for embolization. The patient recovered well after the procedure, with significant mental improvement. This suggests that the deployment of GDCs in the afferent artery near a fistula before embolization with a liquid embolic agent can minimize the risk of uncontrolled penetration of the embolization into the draining veins and dural sinus. A multisession procedure can be an effective and reasonable method of PAVF and DAVF occlusion among existing treatment options.

Intracranial pial arteriovenous fistula (PAVF) is a rare cerebrovascular pathology, which comprises a specific subgroup of arteriovenous shunts and accounts for 1.6 to 4.7% of all intracranial arteriovenous malformations. 1 2 3 4 5

However, unlike other arteriovenous malformations, PAVF does not demonstrate a nidus, is supplied from the pial or cortical arteries, and is characterized by abnormal direct high-flow connection between an arterial feeding vessel and draining vein, which has a significant impact on the cerebral blood flow. 6 7 The high rate of arteriovenous shunting and deep venous drainage significantly decreases the safety and efficacy of microsurgical treatment, and nowadays, endovascular treatment of PAVF is preferable. 8 9 The presentation of disease occurs in early childhood, and it can be present with hydrocephalus, intracranial hemorrhage, seizures, heart failure, mental retardation, and focal neurological signs. 10 Congenital PAVF may have syndromic association with Rendu–Osler–Weber disease and Klippel–Trenaunay–Weber syndrome. 11

A dural arteriovenous fistula (DAVF) is a pathological shunt between the meningeal arteries and dural sinuses or meningeal veins. 12 One of the predisposing factors for DAVF formation is venous hypertension, in which microvascular connections are formed in the dura mater with subsequent transformation into shunts between arteries and veins. 10 13 DAVFs account for 10 to 15% of all cerebral arteriovenous shunts. 12 An association between PAVF and DAVF makes their diagnosis and treatment more challenging. Most authors consider endovascular techniques to be the safest and more effective in treating this pathology. 4 5 6 8

In our present report, we describe a case of PAVF–DAVF in a 2.5-year-old child treated by endovascular method with good clinical outcomes.

## Case Report

**Fig. 1:**
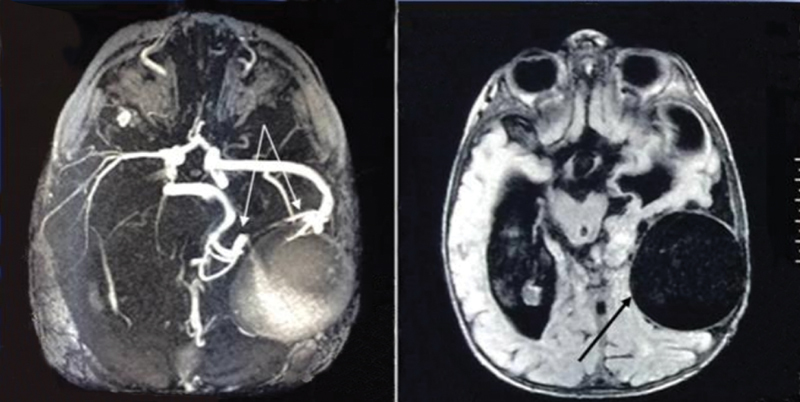
Cerebral magnetic resonance angiography ( **A** ) and magnetic resonance imaging ( **B** ) T1-weighted images demonstrating the intracranial arteriovenous fistula (arrows).

**Fig. 2:**
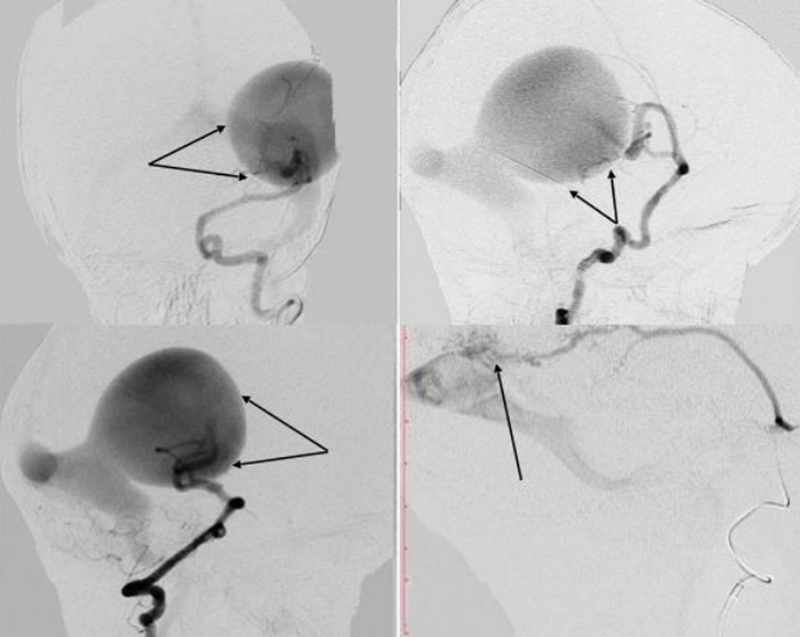
Cerebral angiograms showing an intracranial pial–dural arteriovenous fistula. Anterior-posterior ( **A** ) and lateral ( **B** ) left internal carotid and lateral left vertebral arteries ( **C** ) angiograms showed pial arteriovenous fistula (arrows) with a hypertrophied draining vein. Cerebral angiogram with lateral left middle meningeal artery injections ( **D** ) demonstrated dural arteriovenous fistula (arrow).

A 2.5-year-old male child was admitted to the State Organization “Scientific-Practical Center of Endovascular Neuroradiology, NAMS of Ukraine” in February 2014. Intracranial arteriovenous fistula was diagnosed in utero. The patient was born at term, and up to 1 year of age, showed no mental retardation, and began to walk and speak certain words at 1 year of age. However, after 1 year, a gradual decrease in mental development with progressive vision loss was noted, and 2 months later, first ictus of generalized seizures occurred. Subsequently, the patient stopped walking and talking. At admission, the patient demonstrated hydrocephalus, severe mental retardation, right pyramidal insufficiency, seizures, without any evidence of heart failure. Using magnetic resonance imaging (MRI) and magnetic resonance angiography (MRA), an intracranial arteriovenous fistula was diagnosed ( Fig. 1 ). Further, superselective digital subtraction angiography revealed a PAVF–DAVF. The lesion was “multiple-channel” type with afferents from left middle cerebral artery (two feeding arteries), left middle meningeal artery (DAVF), and left posterior cerebral artery (two feeding arteries) ( Fig. 2 ).

**Fig. 3:**
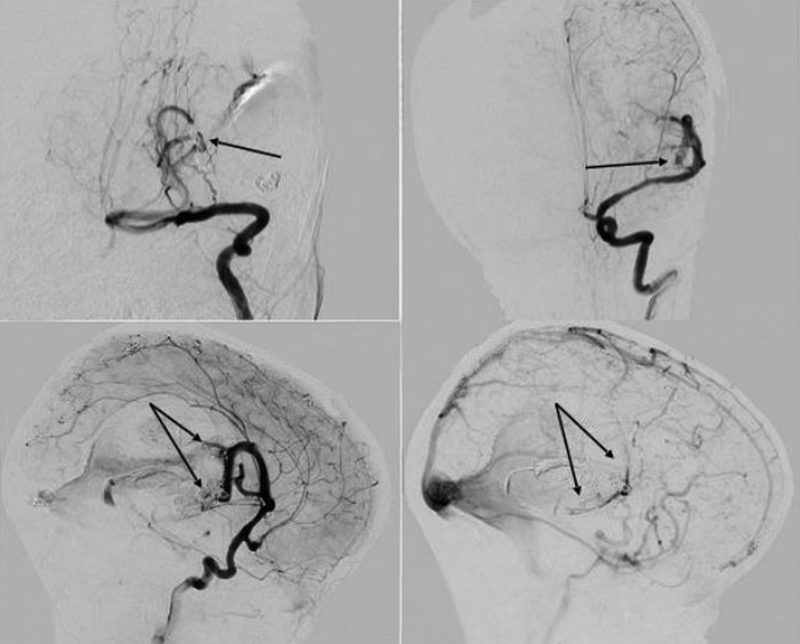
Anteroposterior left vertebral artery ( **A** ) cerebral angiogram demonstrating total occlusion of the pial arteriovenous fistula (arrow). Anteroposterior ( **B** ) and lateral ( **C** ) left internal carotid artery angiograms showing slight penetration of the contrast agent into the aneurysmally dilated draining vein through the partially occluded fistula (arrows). Lateral left internal carotid artery angiogram (late venous phase) ( **D** ) showing contrast retention in the dilated drainage vein (arrow).

Endovascular occlusion of the arteriovenous fistula was performed in two stages, that is, in February and August 2014. The aim of the procedure was disconnection of direct AV shunts. During the first session of PAVF occlusion, the purpose was to slow the blood flow in the fistula; to achieve this, at the level of the fistula entrance in a parent artery, Guglielmi detachable coils (GDCs) (Boston Scientific, CA, USA) were deployed. Then, between the loops of the coils, a liquid embolic agent (Histoacryl; Braun, Melsungen, Germany) with lipiodol (Guerbet, Roissy, France) was injected. After the first stage, total occlusion of the PAVF from the left posterior cerebral artery and incomplete occlusion from the left middle cerebral artery were achieved ( Fig. 3 ). One fistula from the left middle cerebral artery was consciously not occluded because significant contrast retention was observed in the dilated draining vein. To reduce the risk of venous stasis and cerebral venous thrombosis, as well as thrombosis of the significantly dilated draining vein with a significant risk of a mass-related effect, we performed subtotal occlusion of the fistula ( Fig. 3 ).

**Fig. 4:**
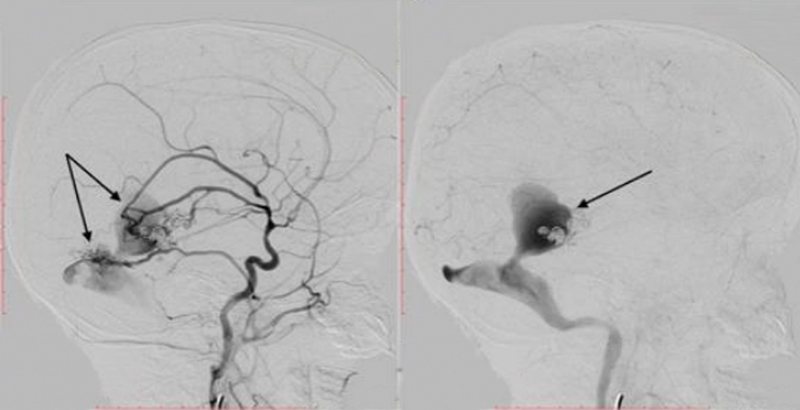
Control angiography after 6 months since the first session of embolization. Lateral left carotid artery angiograms ( **A** , **B** ) reveal dural arteriovenous fistula in the distribution of the left middle meningeal artery ( **A** ), a partially occluded pial arteriovenous fistula ( **A** ) (arrow), and a significant decrease of the draining vein ( **B** ) (arrow).

The patient was admitted to our hospital after 6 months in August 2014 with a significant improvement in neurological state; his vision was also completely restored, and the right-sided pyramidal insufficiency had regressed to mild hemiparesis. The patient began to independently walk and speak. However, control cerebral angiography revealed the persistence of a left middle meningeal artery DAVF and a partially thrombosed left middle cerebral artery PAVF after the first procedure, with a significant decrease in the drainage vein ( Fig. 4 ).

**Fig. 5:**
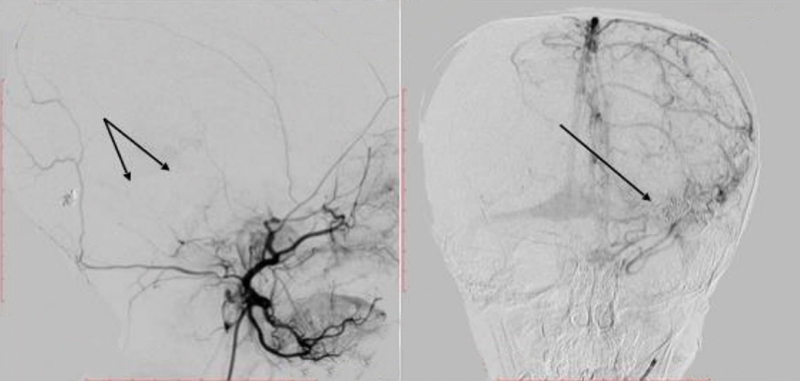
Lateral left external carotid artery angiogram ( **A** ) and anteroposterior left internal carotid artery angiogram (venous phase) ( **B** ) demonstrating total occlusion of pial–dural arteriovenous fistula after the second stage of the surgical procedure (arrows).

During the second session, the shunts of PAVF and DAVF between the left middle meningeal artery and left middle cerebral artery were totally occluded with the aforementioned liquid embolic agent ( Fig. 5 ).

**Fig. 6:**
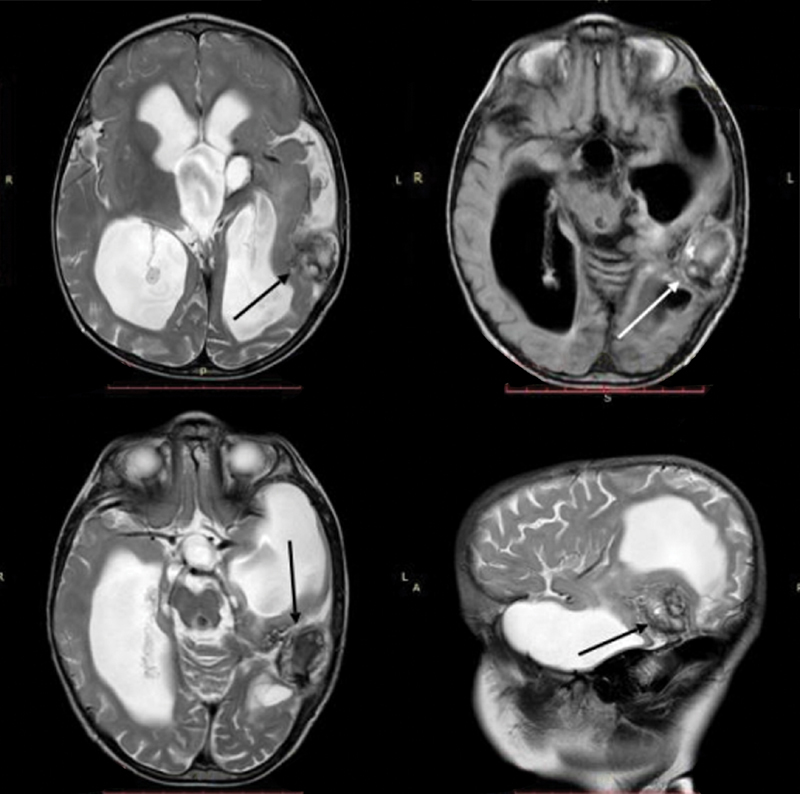
Brain magnetic resonance imaging axial T2-weighted images ( **A** , **C** ), axial fluid-attenuated inversion recovery image ( **B** ), and sagittal T2-weighted image ( **D** ) demonstrate a decrease in the size and signs of thrombosis of the draining vein (arrows) with a reduction in the hydrocephalus after endovascular embolization of pial–dural arteriovenous fistula.

After the second stage of PAVF–DAVF embolization, the patient was discharged with satisfactory results. MRI after 4 months revealed a significant decrease of the previously dilated draining vein and signs of its thrombosis, with a reduction in the mass-related effect on the brain structures and the severity of hydrocephalus ( Fig. 6 ).

Four years after embolization, the patient's neurological status remained unremarkable, and his psychosocial and motor development was age-appropriate.

**Fig. 7:**
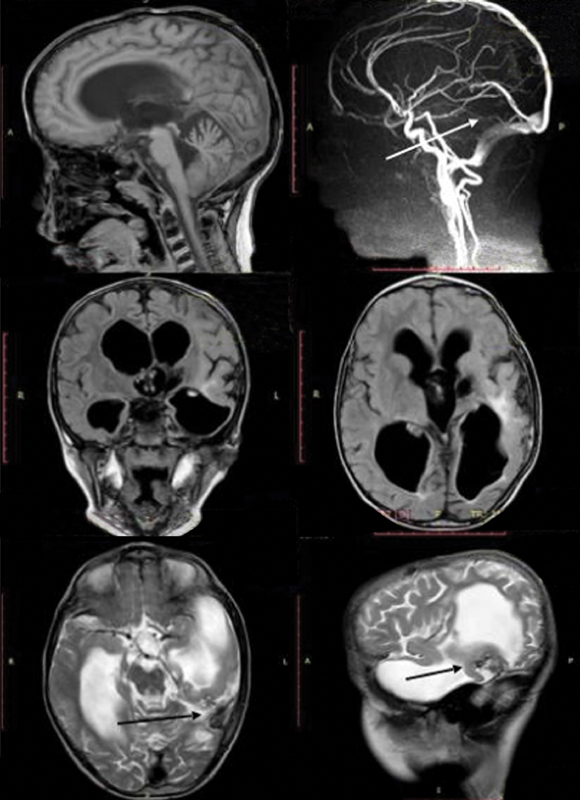
Brain magnetic resonance imaging (MRI) and magnetic resonance angiography (MRA) at 4 years after surgery. Sagittal T1-weighted image ( **A** ) and T2-weighted image ( **F** ), axial fluid-attenuated inversion recovery ( **D** ) T2-weighted image ( **E** ), and coronal FLAIR image ( **C** ) showed a decrease in the hydrocephalus as compared with the first MRI scan taken after the first embolization session and the normalization of the draining veins ( **E** , **F** ) (arrows). MRA sagittal image ( **B** ) shows the complete occlusion of pial–dural arteriovenous fistula (arrow).

Brain MRI and MRA revealed a decrease in the hydrocephalus, total occlusion of the arteriovenous fistula, and almost complete resolution of the previously dilated drainage vein with MRI signs of its thrombosis ( Fig. 7 ).

## Discussion

The manifestation of an intracranial PAVF usually occurs in early childhood and can be present with intracranial venous hypertension with the development in hydrocephalus, epileptic seizures, delayed mental retardation, and focal neurological signs, as observed in this patient at the time of admission to the hospital. 10 Cerebral distal subtraction angiography revealed the detailed angioarchitecture of the intracranial arteriovenous fistula and visualized the combination of the PAVFs and DAVFs.

The treatment of PAVF should include multidisciplinary approach and be based on the experience of the medical team. It is important to evaluate the PAVFs location and degree of lesion complexity. 4 14 PAVFs, with a complex configuration of arteries supply with multiple arterial connections and high flow in noneloquent areas, are cases in which the microsurgical resection has greater benefits. 15 In our opinion PAVF in eloquent areas, without many feeding vessels, can be safely treated using endovascular approach. The endovascular procedure has been considered less invasive and is adopted as the treatment of choice by many authors. Endovascular treatment can be done in a few stages to reduce the risk for postoperative hemorrhage by preventing normal perfusion pressure breakthrough in high-flow lesions.

The treatment strategy in each case has to be made by the team of neurosurgeons and neurointerventionists to identify the best effective method based on patient-specific presentation and angioarchitecture. 16

In our case, a two-staged surgical procedure using a simple and widely available technique (a combination of a liquid embolic agent [Histoacryl with Lipiodol] and GDCs) resulted in the total occlusion of PAVF–DAVF. Cerebral venous thrombosis is a terrible complication after the endovascular embolization of PAVF. Complete occlusion can lead to the extensive thrombosis of the draining veins, causing venous infarction or intracranial hemorrhage. 6 17 It seems that partial embolization performed in the first session was the right decision, less risky, and prevented the abrupt thrombosis of the expanded draining vein, causing fast regression of the neurological deficits in the early postoperative period and a favorable clinical outcome during long clinical and neuroimaging follow-up.

## Conclusion

Understanding the angiographic peculiarities of arteriovenous fistulas angioarchitecture is important for optimal treatment planning. Endovascular embolization in high volume center that is performed by experienced neurointerventionists is a safe and effective technique for treating this pathology. The introduction of GDC at the level of the fistula entrance in a parent artery before the application of the liquid embolic agent can minimize the risk of uncontrolled penetration of the embolization into the draining veins and dural sinuses. A multisession procedure can be an effective and reasonable approach to PAVF and DAVF occlusion among existing treatment options. Ultimately, the elimination of abnormal arteriovenous shunting in children allows the normal development of the central nervous system.
